# Recent updates on cold adaptation in population and laboratory studies, including cross-adaptation with nonthermal factors

**DOI:** 10.1186/s40101-025-00387-6

**Published:** 2025-02-19

**Authors:** Hitoshi Wakabayashi, Hiroyuki Sakaue, Takayuki Nishimura

**Affiliations:** 1 Faculty of Engineering, Hokkaido University, N13 W8 Kita-Ku, Sapporo, 060-8628 Japan; 2 Faculty of Health and Sport Sciences, University of Tsukuba, 1-1-1 Tennodai, Tsukuba, 305-8574 Japan; 3 Faculty of Design, Kyushu University, 4-9-1 Shiobaru, Minami-Ku, Fukuoka, 815-8540 Japan

**Keywords:** Brown adipose tissue, Skeletal muscle, Cold-induced vasodilation, Cold shock response, Habituation, Metabolic adaptation, Insulative adaptation, Cross-adaptation, Exercise training, Hypoxia

## Abstract

This review aims to update our understanding of human cold adaptation. First, an overview of the thermoregulatory response to cold is provided, with some recent updates in human brown adipose tissue (BAT). Variation in BAT activity and multiorgan contributions to cold-induced thermogenesis were introduced. We found that individuals with less BAT activity rely more on shivering to compensate for less non-shivering thermogenesis (NST). The mechanisms of cold-induced vasoconstriction are summarized, including the role of arteriovenous anastomoses, adrenergic neural function, and inhibition of the nitric oxide vasodilator pathway. In addition, cold-induced vasodilation (CIVD) during cold immersion of the distal extremities is summarized with some recent updates in physiological mechanism. Furthermore, the cold shock response at the onset of cold immersion is introduced. Next, categorization of cold acclimatization/acclimation into habituation of shivering and metabolic and insulative adaptation are provided, with some recent updates. Especially, the rediscovery of human BAT has clarified metabolic acclimation, where increased NST replace shivering. Then, a greater CIVD response in populations in cold regions has been reported, whereas recent laboratory studies suggest no increase in CIVD after repeated cold exposure. To prevent cold injuries, individuals should not rely on habituation through repeated cold exposure. In addition, habituation to the cold shock response after repeated cold water immersion could help reduce the number of drownings. Furthermore, cross-adaptation between cold and nonthermal factors in the thermoregulatory response is summarized. Recent studies explored the relationship between exercise training and BAT activity, although this remains unresolved, depending on the exercise intensity and environmental conditions. The effects of exercise with cold exposure on the thermoregulatory response to cold are summarized in studies including divers working in cold water. We investigated the effect of exercise training in cold water, which resulted in increased muscle deoxygenation during submaximal exercise and greater anerobic power. Moreover, the effects of a hypoxic environment on cold adaptation are summarized. Elevated basal metabolism and higher distal skin temperature in highlanders could improve their cold tolerance. Finally, factors affecting cold adaptation are discussed. The type of cold adaptation may depend on the specific thermoregulatory responses repeated during the adaptation process.

## Background

In modern times, much of our daily life is spent indoors, leading to reduced exposure to cold environments and decreased physical activity. However, during the Great Journey out of Africa, as humans moved into high-latitude regions, they needed physiological and genetic adaptations to cold environments. There has been a long history of research on cold adaptation in humans through field studies focusing on the responses of indigenous populations in cold regions, as well as laboratory studies evaluating adaptive changes following repeated cold exposure. However, the recent rediscovery of human brown adipose tissue (BAT) activity in mild cold environment, which is also linked to metabolic disease in today's inactive populations, could have an impact strong enough to transform the conventional interpretation of those previous findings. Thus, this article aims to identify research gaps between prior knowledge and recent discoveries to update our understanding of human cold adaptation. Additionally, it has been considered that the types of acquired cold adaptation are influenced directly or indirectly by nonthermal factors such as lifestyle and physical activities. However, these factors have not been sufficiently examined. In this review, we focus on cross-adaptation between cold and nonthermal factors, particularly the effects of exercise and hypoxia on the adaptive thermoregulatory response in cold environments, for better understanding how these factors interact and contribute to cold adaptation. Through the investigation on the cross-adaptation in today’s sedentary population, this review seeks to provide insights into the inherent adaptability of humans to cold environments.

## Physiological responses to cold environments

To maintain core body temperature (*T*_co_) within a certain range in response to changes in the external environmental temperature, the human body produces autonomic thermoregulatory responses such as vasoconstriction/vasodilation, metabolic heat production, and sweating. The point at which the environmental temperature decreases and at which cold-induced thermogenesis (CIT) begins is called the thermogenic threshold (lower critical temperature), and the point at which the temperature increases and the thermoregulatory sweat response begins is the sweating threshold (upper critical temperature). The temperature range is located between the thresholds of CIT and sweating, where heat dissipation is regulated mainly by the skin vasoconstriction/vasodilation response (cutaneous vasomotor regulation zone), which is called the inter-thresholds zone or the thermoneutral zone. The center regulating the balance between heat dissipation and heat production through these thermoregulatory responses exists in the hypothalamus of the diencephalon. It controls cutaneous blood vessels and BAT, white adipose tissue, liver, skeletal muscle (SM), sweat glands, and other effector organs in a complex manner via the sympathetic nervous system, somatic nervous system, and endocrine system.

In this review, we first introduce CIT and cold-induced vasoconstriction, which are autonomous thermoregulatory responses to cold environments below the thermogenic threshold, that is, in the metabolic regulation zone (chemical control zone). In addition to the general thermoregulatory responses for maintaining *T*_co_, this review includes a literature review on cold-induced vasodilation (CIVD), which is observed when hands and fingertips are immersed in cold water. Moreover, we introduce cold shock responses, which are vigorous ventilatory and cardiovascular initial responses when the whole body is immersed in cold water.

### Thermoregulatory response in cold

#### Cold-induced thermogenesis

##### Shivering and non-shivering thermogenesis

**Fig. 1:**
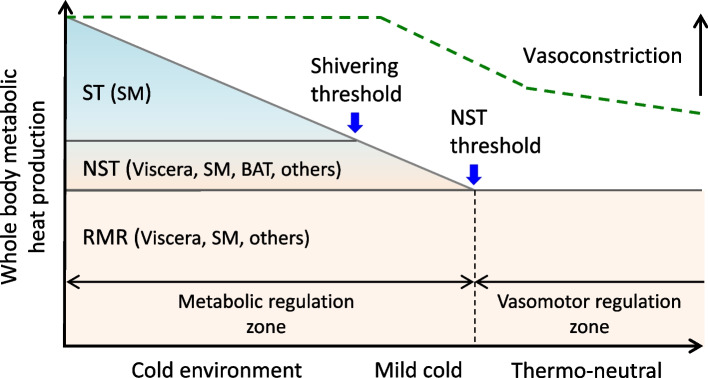
Scheme of the autonomic thermoregulatory response in a cold environment―Non-shivering and shivering components of cold-induced thermogenesis with major thermo-effector organsー ST: shivering thermogenesis, NST: non-shivering thermogenesis, RMR: resting metabolic rate, SM: skeletal muscle, BAT: brown adipose tissue. [1]

In cold environments, maintaining *T*_co_ in humans enhances the CIT, which can be divided into non-shivering thermogenesis (NST) and shivering thermogenesis (ST). Figure 1 shows a schematic diagram of the relationship between the thermal environment and components of metabolic heat production, with major thermogenetic tissues for each component [1].

In a thermoneutral environment, basal metabolism and resting metabolism occur in visceral tissues such as the liver and SM. The basal metabolic rate (BMR) and resting metabolic rate (RMR) are classified as obligatory NST and are sometimes distinguished from adaptive thermogenesis, such as cold-induced NST. In mild cold environments below thermoneutral zone, cold-induced NST occurs in tissues such as BAT, SM, and internal organs. In this scheme of CIT (Fig. 1), the thermogenic threshold at which cold-induced NST begins to occur is specifically defined as the NST threshold. Then, in a cold environment, the ST is generated in the SM, and the threshold at which shivering begins is defined as the shivering threshold. Previously, the thermogenic threshold of the human thermoregulatory response was often used synonymously with the shivering threshold [2, 3]. Recently, since the rediscovery of human BAT activity in 2009 [4–6], researchers have revised the model to distinguish the NST and ST components separately with each threshold [7].

There are large individual variations in NST enhancement during mild cold exposure, ranging from a few percent to 30% of the RMR [7]. Our research group also evaluated NST in a climatic chamber at 18.6 °C in young Japanese males and reported individual variations in NST; the higher the BAT activity was, the greater the increase in NST was, ranging from almost no change to approximately 30% RMR (13.5% on average) [8]. In a study with a larger number of participants, it was reported that in a mild cold environment of 19 °C, NST increased by approximately 5% RMR in individuals with low BAT activity and approximately 18% in individuals with high BAT activity [9].

ST during cold exposure refers to heat production due to involuntary muscle tremors for thermoregulation induced by a decrease in skin and core body temperature. The maximum ST reaches approximately five times the RMR and 40 to 50% of the maximal oxygen uptake [10]. The amount of heat generated by ST is considerably greater than that generated by NST, since SMs are widely distributed throughout the body. Electromyography (EMG) is often used to objectively evaluate the onset of shivering and shivering intensity. During the early phase of cold exposure, muscle tonus and continuous mild tremors occur, and the recruitment of low-threshold motor units (slow-twitch muscle fibers) is observed in the EMG waveform [11]. Afterwards, burst-like high-intensity shivering caused by higher-threshold motor units (fast-twitch muscle fibers) occurs [10, 11], which are mixed with continuous low-intensity shivering. As the intensity of shivering increases, the frequency of burst-like shivering increases, and carbohydrate metabolism increases [10]. There are individual differences in the location of muscle tremors, but the shivering intensity evaluated by EMG is greater in the trunk and lower in the extremities [10, 11].

Although the schematic diagram divides CIT into components of NST and ST by demonstrating the major thermo-effectors, the contribution ratios of BAT and SM for NST are not specified (Fig. 1). With respect to the contribution of BAT to NST, some studies have reported that the contribution of BAT is negligible [12, 13], whereas others have reported a greater contribution of BAT [14, 15]. In addition, because BAT activity exhibits inter-/intra-individual variations, such as group differences, individual differences, and seasonal and diurnal variations [16], it is not possible to uniformly determine the average contribution of each tissue to NST. The next section summarizes the contribution of each tissue to NST according to individual differences in BAT activity and its relationship with other thermoregulatory responses.

##### Inter- and intra-individual variation in BAT activity

Before explaining group and individual differences in BAT activity, the standard BAT evaluation methods are briefly outlined here. Currently, FDG-PET/CT, which combines positron emission tomography (PET) and computed tomography (CT) to image the uptake of ^18^F-fluorodeoxy glucose (FDG) into BAT during mild cold exposure, is most commonly used [17]. The mean standard uptake value (SUV_mean_) and maximal standard uptake value (SUV_max_) in the region of interest, where BAT is present, are analyzed and used as an index of BAT activity and as a threshold for determining BAT-positive or -negative individuals. On the basis of BAT activity during mild cold exposure conducted in several countries, the detection rate of BAT-positive individuals reportedly differs depending on the population [18]. Compared with the high detection rates of 95% in the Netherlands (Maastricht) and 70% in the Finnish (Turku) population, the detection rates of 41% in the Japanese (Sapporo) [19] and 36% in the American (Detroit) populations were relatively low. Although these population differences include the influence of the environments where participants live and the protocols used in each research group, previous studies have compared South Asian and European populations living in the same region via the same evaluation technique [20]. They evaluated BAT activity in South Asian (both parents are from South Asia) and Dutch Caucasian populations born and grown in the Netherlands. Although no group differences were found in the SUV_mean_ or SUV_max_, the BAT volume was smaller in South Asians than in Caucasians [20].

With respect to individual differences in BAT activity within a certain population, early studies reported a relationship between obesity and BAT activity [4–6], and it is well known that obese people have low BAT activity. There is a relationship between type 2 diabetes and BAT activity [21] and between the high prevalence of type 2 diabetes and low BAT activity, especially in South Asian populations [20]. BAT activity is clearly lower in type 2 diabetic patients than in young healthy individuals [22, 23], whereas there is no difference compared with healthy individuals of the same generation [22]. In addition, it is well known that BAT activity decreases with growth and aging [4, 19], and there are also reports of differences depending on outdoor temperature and sex [4, 21].

Variations in BAT activity, such as seasonal differences and circadian rhythms, are observed even within the same individual [16]. BAT activity is greater in winter, when the outdoor temperature is low, than in summer [5, 24]. In addition, repeated short-term cold exposures increase BAT activity in mild cold environments [9, 15, 25, 26]. Furthermore, in a recent study, we reported diurnal variation in BAT activity during mild cold exposure [27]. The NST assessed by the expired gas and supraclavicular skin temperature as a surrogate measure for BAT thermogenesis tended to be greater in the morning and lower at night in those with high BAT activity, whereas no diurnal differences were observed in those with low BAT activity [27]. This suggests a diurnal variation in BAT activity [27].

As described above, since there are inter-individual and intra-individual variations in BAT activity [16], the contribution of BAT to NST also varies and fluctuates. Owing to the variation in BAT activity, SMs and other organs have diverse degrees of contribution as thermogenic effectors. In the following paragraphs, the metabolic contributions of multiple organs to the CIT, including the NST and ST, are summarized.

##### BAT and SM multiorgan contributions to CIT

**Fig. 2:**
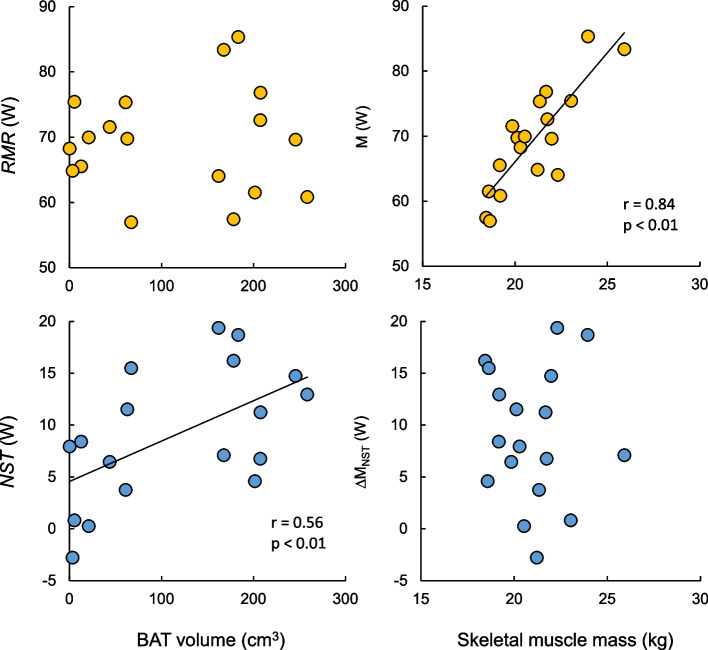
Metabolic heat production during cold exposure as a function of BAT volume and skeletal muscle mass RMR: resting metabolic rate, NST: non-shivering thermogenesis, BAT: brown adipose tissue, SM: skeletal muscle. Modified from Wakabayashi et al. [2020a]

BAT activity and detection rates are particularly low in Asian populations compared with European populations [18, 20]. Thus, there may be greater variation in BAT activity and multiorgan metabolic contributions to CIT in Asian populations. Therefore, we investigated the relationships between the CIT during mild to moderate cold exposure and the amount of BAT and SM in young Japanese males [8]. Starting from the thermoneutral baseline measurement of the RMR at 28 °C in a climate chamber, the room temperature was controlled at a mild cold environment of 18.6 °C for 90 min to induce NST, and then the temperature was further lowered to 11.6 °C to initiate shivering. The main results revealed a positive correlation between NST and the detectable volume of bioactive BAT, which was assessed via the FDG-PET/CT technique, but there was no relationship with the SM mass (Fig. 2). The contribution of BAT to NST is consistent with the findings of previous BAT studies [6, 9, 12, 13]. On the other hand, although there was a strong positive correlation between SM mass and RMR, no contribution of SM to NST was observed (Fig. 2). This finding is consistent with the finding that lean body mass was positively correlated with RMR but not with NST in supplemental data from a previous study [9]. These results suggest that the contribution of SM to NST may be smaller than that of BAT; however, there is a limitation in the evaluation of the contribution of SM to NST based only on the estimated tissue weight without any measurement of bioactive muscle metabolism.

Several research groups have argued for the high contribution of SM to thermogenesis during mild cold exposure on the basis of the assessment of tissue metabolism via FDG-PET and [^15^O]O_2_-PET [13, 28]. A study evaluating FDG uptake in multiple organs during mild cold exposure (including minimal shivering) revealed that although glucose uptake per unit volume was greater in BAT than in SM, total glucose uptake considering tissue volume was much greater in SM [28]. However, the total glucose uptake shown in this study included the RMR component, and the contribution of both tissues to the NST component was not evaluated. In our study, the total metabolic rate combined with the RMR and NST during mild cold exposure was positively correlated with the SM mass (*r* = 0.683, *P* < 0.01), but this was mostly due to the component of the RMR, which was strongly correlated with the SM mass. In a study in which [^15^O]O_2_-PET was used to evaluate oxygen uptake in each tissue during mild cold stimulation, the tissue-specific NST (difference in oxygen uptake during cold stimulation and at a room temperature of 22 °C) in the BAT around the cervical and upper thoracic regions was only approximately 1% of the total NST in the whole body and was not correlated with the total NST. However, when tissue-specific NST from SMs around the cervical and upper thoracic regions are combined, there is a positive correlation with whole-body NST [13]. These results suggested that the contribution of BAT to total NST was small, and that SM was the main thermogenic tissue for NST. Similarly, some studies have reported that energy expenditure in BAT is approximately 5% of total whole-body NST on the basis of evaluation of tissue oxygen uptake via the [^15^O]O_2_-PET technique [12]. Although the evaluation of the contribution of each tissue metabolism to CIT via [^15^O]O_2_-PET is useful and important, this technique might lead to underestimation of BAT-specific metabolism because of the short half-life of [^15^O] of approximately 2 min and the limited area of view [14]. Indeed, according to a study using FDG-PET/CT [15], a greater contribution of BAT to NST was suggested, with large individual variation from almost 0 to 16% RMR accounting for 90% of NST [14].

With respect to the contribution of SM to NST according to BAT activity, specificity can be seen in the adaptive phenomena of each tissue’s metabolism caused by repeated cold exposure. In young healthy individuals with high BAT activity, repeated cold exposure induced an increase in NST and increased BAT activity (increased FDG uptake), whereas no adaptive change was observed in FDG uptake into the SM [25] or in parameters of SM respiration, such as mitochondrial density and respiration [15]. On the other hand, studies targeting type 2 diabetic patients and obese individuals with low BAT activity have shown that repeated cold exposure not only increases BAT activity but also increases glucose uptake into the SM [23, 29]. Considering these findings, in populations with low BAT activity, since the contribution of SM to NST is relatively high, tissue-specific metabolic adaptation due to repeated cold exposure might be enhanced in SM. Further research is needed to clarify the degree of contribution of each organ to NST, as well as its diversity and fluctuations.

**Fig. 3:**
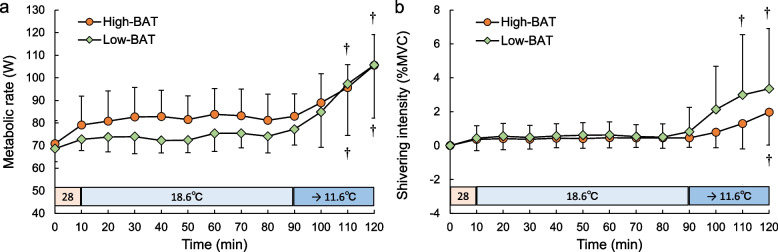
Time course of metabolic rate (**a**) and shivering intensity (**b**) during gradual cold exposure in the high- and low-BAT activity groups The values are the means±SDs. † Significant difference compared with values at the end of mild cold exposure (90 min) in each group. Modified from Wakabayashi et al. [2020a]

**Fig. 4:**
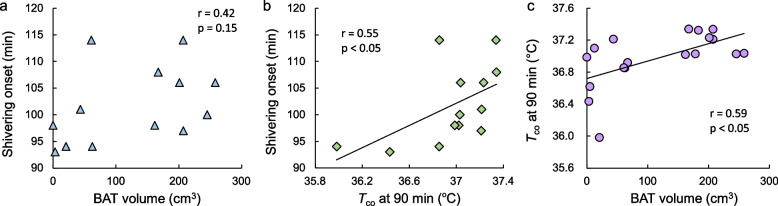
Relationships between BAT volume, core body temperature, and shivering onset time Modified from Wakabayashi et al. [2020a]

In our study, in addition to examining the contributions of BAT and SM to NST during mild cold exposure (18.6 °C), we also examined the relationships between NST and shivering induced by a further reduction in the ambient temperature to 11.6 °C [8]. When the participants were divided into high- and low-BAT activity groups, the high-BAT activity group presented a greater metabolic rate during the mild cold exposure phase (Fig. 3a). During the subsequent phase of shivering onset, the shivering intensity of the pectoralis major muscle, as assessed by EMG and expressed as the percentage of maximal voluntary contraction (MVC) [30], increased earlier in the low BAT activity group than in the high BAT activity group (Fig. 3b). After 90 min of mild cold exposure, when the environmental temperature was lowered to 11.6 °C, although no relationship was observed between the shivering onset time and the amount of BAT (Fig. 4a), a negative correlation was shown between the *T*_co_ at 90 min and the time of shivering onset (Fig. 4b). This could be because the greater the amount of BAT was, the greater the NST was, and the higher the *T*_co_ was (Fig. 4c); as a result, the onset of shivering was delayed. Although a direct relationship between BAT activity and ST by SM was not clearly observed, there might be an indirect correlation between BAT-mediated NST and SM-mediated ST for maintaining *T*_co_.

In a study comparing CITs between South Asian and Caucasian populations, an individualized cooling protocol was used to detect the shivering threshold, after which the cold stimulus was adjusted to assess BAT activity at maximum NST [20]. As mentioned above, the South Asian group presented a lower BAT content and less NST; in addition, the shivering threshold temperature was approximately 2 °C higher on average than that of the Caucasian population [20]. In other words, in South Asian populations with low BAT activity, relatively mild cold stimulus causes the onset of shivering to compensate for less NST. Similarly, in type 2 diabetic patients with low BAT activity, in addition to higher glucose uptake in SM than in young healthy participants, these patients tended to have higher shivering intensity (%MVC) during mild cold exposure [22]. Moreover, there was a tendency for a negative correlation between BAT volume and shivering intensity in all participants, and those with low BAT activity might induce somewhat stronger shivering in SM [22].

These previous studies focused mainly on NST, and only minimal shivering was observed during mild cold exposure [20, 22]. In our study, the shivering phase was only 30 min at 11.6 °C, which was an insufficient duration and cold stimulus for initiating strong shivering or reaching a steady state [8]. It is necessary to further investigate the multiorgan compensation in metabolic thermogenesis between SM-induced ST and NST according to the variation in BAT activity.

#### Cold-induced vasoconstriction

To maintain *T*_co_ in a cold environment, in addition to the CIT for increasing heat production, cutaneous vasoconstriction is also induced for heat conservation. Many studies have been conducted on vasomotor thermoregulation, including vasoconstriction and vasodilation, which have been summarized in several review articles [31–34]. In the following paragraph, an overview of the vasomotor response to cold is provided.

Vasomotor regulation is initiated even within the thermoneutral zone where the cold stimulus is milder than the NST threshold (Fig. 1). The cold-induced cutaneous vasoconstriction decreases *T*_sk_ and reduces the temperature gradient between the skin and surrounding environment, which results in the suppression of dry heat loss from the skin surface. When cutaneous vasoconstriction occurs, venous return from the extremities shifts from superficial to deeper veins [35]. This enables the human body to reduce heat loss since deeper venous veins are well insulated compared with superficial veins. In addition, countercurrent heat exchange occurs between the deep veins (accompanying veins) and arteries that run parallelly and intermingle in opposite blood flow directions [36], and the temperature of the arterial blood gradually decreases, resulting in a lower *T*_sk_ at the distal extremities. Thus, a nonuniform *T*_sk_ distribution in distal body regions is often observed in cold environments. From a morphological perspective, since the surface area per volume is greater in the distal extremities, particularly in the fingers and toes, than in the trunk body, lowering the *T*_sk_ at the distal extremities is a rational way to reduce dry heat loss.

From a vascular anatomical perspective, arteriovenous anastomoses (AVAs) are located in the skin of distal body parts, especially in the fingers, toes, and glabrous (acral) sides of the hands, feet, ears, and nose, but not in the trunk body parts [37]. AVAs have a structure of direct connection between small arteries and veins, without capillary segments for exchanging substances with tissues. The length of AVA typically ranges from 200 to 500 μm, the inner diameter ranges from 10 to 150 μm, and the thickness of the wall ranges from 40 to 60 μm, with an inner layer of smooth muscle cells, which enables them to close the vessels completely [37]. Since the amount of blood flow through the vessels is proportional to the 4th power of its inner diameter according to Poiseuille’s law, compared with ordinal capillaries with a 10-μm inner diameter, 256 times greater blood flow should pass through the AVA with a 40-μm inner diameter [38] and over 1000 times greater blood flow through the AVA with a 60-μm diameter [39]. In the case of AVAs 100 μm in diameter, the amount of blood flow could reach 10,000 times greater than that in capillaries. These anatomical properties enable AVAs to have very high blood flow and heat transfer from the body core to the skin surface. It has been suggested that AVAs are regulated by adrenergic sympathetic nerve activity [37, 39]. Indeed, very heavy innervation of noradrenergic fibers was observed around AVAs [40]. This finding is supported by physiological evidence that fluctuations in blood velocity in the dorsalis pedis artery disappear after epidural block [41]. In addition, the rapid vasoconstriction and slower vasodilation process indicate that vasoconstriction is induced by burst impulses in the adrenergic axons innervating the AVAs, and that vasodilation is passively induced by the release of AVA smooth muscle contractions [37].

The AVAs located in the acral skin play a major role in heat dissipation from the skin within the thermoneutral zone. When the ambient temperature is below the thermogenic threshold (lower edge of the thermoneutral zone), all AVAs remain closed [37]. The effect of moderate local cooling on blood velocity in the finger artery was examined with hand immersion from 35 to 19 °C water [42], and sustained closure of AVAs was observed when the water temperature (*T*_w_) was below 21.5 °C (ranging from 20 to 23 °C). However, with severe local cooling of the finger in 3 °C water, the same research group observed cold-induced vasodilation (CIVD) in the blood velocity in the finger arteries [43]. On the basis of the time course data, they suggested that dilation of AVAs, which could be due to relaxation of low-temperature smooth muscle cells, is a mechanism eliciting CIVD [43].

The physiological mechanism of cold-induced vasoconstriction in non-glabrous (non-acral) skin parts is briefly summarized below [33, 34]. Whole-body cooling induces cutaneous vasoconstriction, even if the site of the skin blood flow (SkBF) measurement is not locally cooled, and this reflex cutaneous vasoconstriction is controlled by the sympathetic nervous system [33]. Noradrenaline (NA) released from adrenergic nerve endings is a primary neurotransmitter that binds with adrenergic receptors (or adrenoceptors) on vascular smooth muscle to induce vasoconstriction [33, 34]. In addition, neuropeptide Y (NPY) is known as a major co-transmitter for reflex vasoconstriction when the whole-body *T*_sk_ gradually decreases to 31.7 °C [44]. Another study reported the contribution of adenosine triphosphate (ATP) as a co-transmitter for reflex cutaneous vasoconstriction, which was observed when the whole-body *T*_sk_ gradually decreased to 30.5 °C [45]. It is generally suggested that the NA is responsible for 60% of reflex cutaneous vasoconstriction [33, 45], and the remaining 40% is explained by the co-transmitters such as NPY and ATP; however, further studies are needed to clarify their contributions.

The mechanism of the vasoconstrictor response to local skin cooling varies depending on the duration of cooling [32]. During the early phase of local cooling (within 10 min), vasoconstriction is regulated mostly by adrenergic neural mechanisms, which depend primarily on alpha-2 (*α*_2_) adrenergic receptors [46]. Local cooling increases the amount of reactive oxygen species (ROS) generated from mitochondria in vascular smooth muscle, which enhances Rho-kinase activity [47]. Mitochondrial ROS increase Rho-kinase activity to elicit the translocation of *α*_2c_ adrenergic receptors to the vascular smooth muscle cell membrane [48]. The increased sensitivity of *α*_2c_ adrenergic receptors, through the above process, enhances vasoconstriction, even though NA secretion from the sympathetic nerve is reduced by local cooling [32].

During the late phase of local cooling (more than 10 min), vasoconstriction in non-glabrous (non-acral) skin is regulated by a combination of continued activation of adrenergic function and inhibition of the nitric oxide (NO) vasodilator pathway [32, 49]. NO is a vasodilator substance synthesized by the nitric oxide synthase (NOS) enzyme to relax vascular smooth muscle. This occurs through the process of activating guanylate cyclase, which increases cyclic guanosine monophosphate (cGMP) and activates protein kinase G (PKG), ultimately leading to smooth muscle relaxation. Prolonged local skin cooling inhibits the activity of the endothelial NOS enzyme downstream of NOS enzymes, which suppresses NO-mediated vasodilation, resulting in vasoconstriction [49]. The inhibition of NOS activity is partly induced by the elevation of ROS from vascular smooth muscle mitochondria, which enhances Rho-kinase activity [32, 47, 50]. Since the Rho-kinase pathway is relatively slow, as shown by the time course of Rho activity to cooling in human cultured dermal arteriolar vascular smooth muscle [48], Rho-kinase plays an important role in vasoconstriction during prolonged cooling via both inhibition of the NO pathway and increased sensitivity of *α*_2c_ adrenergic receptors.

### Cold-induced vasodilation

When fingers or hands are exposed to cold water, cold-induce vasoconstriction reduces finger skin temperature (*T*_fing_) and remains low during the early phase of immersion; then, after a while, finger blood vessels vasodilate to increase *T*_fing_. This response is known as CIVD, which was originally discovered by Lewis in 1930 [51]. The CIVD and vasoconstriction are repeated during cold exposure to show the hunting reaction in the time course of *T*_fing_. The paradoxical vasodilation response in cold fingers has been studied for a century, and several review articles have summarized the findings with perspectives for future research [52–54]. Since the upregulation of* T*_fing_ is reasonable for preventing frostbite and nonfreezing cold injury (NFCI), the CIVD response is used as an index of local cold tolerance.

Several parameters of the CIVD response have been defined in previous research [52, 53, 55]. The onset time is the duration from finger immersion to the minimum temperature (*T*_min_), which is induced by the initiation of the first CIVD response. The peak time is the time interval from *T*_min_ to the first maximal temperature (*T*_max_), and the amplitude is the temperature difference between* T*_min_ and *T*_max_. In some cases, the following CIVD waves have lower nadirs or higher peak temperatures than the *T*_min_ or *T*_max_ observed in the first wave. It would be reasonable to choose the first *T*_min_ and *T*_max_, corresponding to the onset and peak times, respectively, while it would depend on the studies and CIVD waves. The mean finger temperature (*T*_mean_) from CIVD onset (in practical use, 5 min after immersion) to the end of cold immersion can be used as a representative parameter for the magnitude of the series of CIVD responses. In addition, the area under the curve from the onset of CIVD to the end of immersion can reflect both the rapidity and magnitude of CIVD [56]. The number of hunting reactions is also used as a parameter of CIVD. There are individuals who do not show any CIVD response. Notably, the changes in the finger SkBF measured by laser Doppler flowmetry precede the changes in *T*_fing_ during the CIVD response [43, 57, 58]. The time lag between the vasomotor response and subsequent *T*_fing_ should be considered, especially when focusing on the timing of the CIVD response and synchronization with other factors.

The potential physiological mechanism of the CIVD response has been summarized on the basis of several review articles [52, 54] with some recent updates. Since the original research on CIVD [51], the axon reflex has been hypothesized as a potential mechanism of the response, which might lead to the inhibition of the sympathetic nerve to AVAs and cause relaxation of smooth muscle. However, this hypothesis was rejected by a study reporting that the axon reflex induced by painful electrical stimulation increased skin blood perfusion in warm fingers but not in cold fingers [59].

As explained above, AVAs have very high blood flow to transfer heat and are located at the distal and acral skin parts where the CIVD response is mainly observed [37]. In addition, on the basis of the blood velocity measurements in the finger arteries with severe local cooling, the CIVD response may reflect relaxation of the AVA smooth muscle due to the low temperature of the smooth muscle cells (local phenomenon), since sustained vasoconstriction was observed in the non-cooled control fingers, indicating no change in the sympathetic impulses [43]. Nervous blockade of the neuromuscular junction from adrenergic nerves to smooth muscle could be induced by low tissue temperature [52, 53]. The cessation of transmitter release from adrenergic nerve endings may regulate CIVD, since no CIVD response was observed in isolated rat tail arteries when the NA was continuously perfused [60]. Cold-induced vasoconstriction limits blood flow to finger vessels, resulting in a critical low tissue temperature for the cessation of NA release from nerve endings. After a while, smooth muscle is relaxed, and CIVD occurs to supply blood and heat to the vessels, which warms the tissue and resumes sympathetic transmitter activity to induce subsequent vasoconstriction. The repetition of the series of vasomotor responses forms the hunting reaction in the finger blood flow and *T*_fing_. In addition to the local phenomenon, it was suggested that sympathetic activity (central phenomenon) could be a major factor for controlling the initiation and magnitude of CIVD, on the basis of the findings of suppressed CIVD with lower *T*_co_ and *T*_sk_ [61, 62], which are associated with high sympathetic activity.

The contribution of some vasodilating substances, such as NO, has been hypothesized as a potential mechanism of CIVD [52–54]. To the best of our knowledge, no clear evidence of the contribution of NO to the human CIVD response has been reported, whereas a study reported that cooling potentiated greater NO production in response to cholinergic stimulation in the cutaneous artery dissected from the rabbit ear [63]. As explained above, prolonged local skin cooling inhibits the activity of endothelial NOS and downstream of NOS enzymes, which suppresses NO-mediated vasodilation, resulting in vasoconstriction in non-glabrous skin [49]. Recently, several updates have been conducted on the mechanism of CIVD based on wavelet analysis of finger SkBF measured via laser Doppler flowmetry [57, 64, 65]. The frequency bands and associated vasomotor functions are 0.005–0.01 Hz (endothelial NO-independent activity), 0.01–0.02 Hz (endothelial NO-dependent activity), 0.02–0.06 Hz (neurogenic activity), and 0.06–0.15 Hz (myogenic activity), with slight variation among studies. During hand immersion in 8 °C water, strong synchronization was observed between the time course of neurogenic activity and laser Doppler flow, whereas both endothelial NO-independent and NO-dependent activities were not synchronized with the SkBF [57]. During finger immersion in 5 °C water, the suppression of neurogenic and myogenic activities and an increase in endothelial NO-independent activity were observed just prior to CIVD, whereas no change in endothelial NO-dependent activity was detected [64]. Although there is some discrepancy in NO-independent endothelial activity between studies, the minor contribution of NO to the CIVD response and the major role of neurogenic activity (sympathetic withdrawal) were confirmed consistently [57, 64]. There are studies suggesting that NO does not contribute to the regulation of CIVD because of the effect of acute supplementation of beetroot (red beet) juice with a high dose of nitrate, which supplies exogenous NO through the sequential reduction process [58, 66]. No effect of beetroot intake on the CIVD parameters was observed in* T*_fing_ and SkBF during hand cold immersion [58, 66], although our study revealed that the vasodilator effect of beetroot enhanced the recovery of *T*_fing_ and SkBF after the hand was removed from cold water [58].

### Cold shock response

The cold shock response (CSR) evoked during the initial few minutes of cold water immersion is a life-threatening reflex that includes cardiovascular (tachycardia, arrhythmia, peripheral vasoconstriction, and hypertension) and respiratory (gasping and hyperventilation) components [67–71]. It has been reported that the CSR begins at *T*_w_ approximately 25 °C, while it can be consciously controlled at that temperature [72], and the respiratory frequency peaks when *T*_w_ is between 10 and 15 °C, which is as high as that in 5 °C water [72, 73].

Tachycardia within a few seconds of exposure to cold water is one of the most frequently reported cardiac CSRs [69]. Exposure to cold water (ice‒cold shower) increases cardiac output and peripheral vasoconstriction, resulting in an increase in arterial pressure within a few seconds and reaching maximal levels within 30–60 s [68]. Several studies have also reported that cold water immersion produces an inspiratory gasp followed by uncontrollable hyperventilation, indicated by a significant decrease in the end-tidal partial pressure of carbon dioxide [67, 74]. Compared with the pre-immersion baseline, cold shock hyperventilation can increase the respiratory rate by more than 110% and the ventilation volume by more than 600% [70, 75, 76], increasing the risk of involuntary water aspiration and drowning [75, 76]. A reduction in the maximal breath hold time caused by the inspiratory gasp response has been reported in several studies [76, 77].

The World Health Organization (WHO) estimates that there were more than 2.5 million drowning deaths between 2008 and 2018 [78]. In the case of accidental cold water immersion, the initial CSR could be one of the major causes of near-drowning incidents and drowning death [79] since initial inspiratory gasping and uncontrollable hyperventilation induce the inhalation of water into the lungs when the head is submerged below the water surface. In addition, during cold water submersion, CSR and the diving reflex are simultaneously induced, and both sympathetically mediated tachycardia and parasympathetically mediated bradycardia could cause “autonomic conflict,” resulting in arrhythmias [80, 81]. A policy of “float first” during the initial minutes of accidental cold water immersion to keep the airway above the water surface is a reasonable practice for preventing the inhalation of water and improving survival prospects [82, 83]. In addition, the habituation of the CSR by repeated cold water immersion, which is discussed later, has the potential to reduce the number of drownings.

The immediacy of the CSR suggests that the components are driven by stimulation of cutaneous cold receptors upon immersion and are mediated by the sympathetic autonomic nervous system [67, 69, 70, 84]. A greater CSR was reported under whole-skin exposure conditions than under partially insulated conditions with a wet suit [76, 85], and a greater CSR was induced during one-stage immersion than during two-stage entry [75]. These findings suggest that the CSR is induced by both spatial and temporal summation of regional thermo-afferent inputs. A study reported that a peripheral cold stimulus to the trunk body part resulted in a greater influence on the ventilatory CSR than when it was applied to other parts of the body [86].

It has been hypothesized that thermo-afferents from peripheral cold receptors increase the ventilatory response by directly stimulating the respiratory center [67, 87]. A chemoreceptor pathway, which is normally activated by higher partial pressures of carbon dioxide and results in an increase in ventilation, does not affect the ventilatory CSR. This was confirmed in a study that reported no difference in ventilatory CSR between conditions with and without hyperventilation prior to cold water immersion [73]. The neural pathways of ventilatory CSR, which are largely based on animal studies, are summarized in several review articles [70, 71].

The cardiovascular CSR is thought to be due to reflex stimulation of the sympathetic nervous system as a direct consequence of rapid skin cooling [68, 88]. Johnson et al. (1977) reported that the level of plasma noradrenaline from the sympathetic nervous system almost doubled after 2 min of immersion in 10 °C water. In contrast, Keatinge et al. (1964) reported no evidence of the release of catecholamines during 2 min in ice–water showers. Cold stimulus to the skin surface of the feet during cold immersion was found to increase beta-adrenergic sympathetic activity to the heart and likely inhibited parasympathetic activity, resulting in tachycardia [89]. More information on the mechanism of CSR is summarized in some review articles [70, 71].

## Cold adaptation

There are several terms that explain cold adaptation, such as “acclimatization,” “acclimation,” and “habituation.” These terms are often used in mixtures, but in this review, the terms follow the glossary of terms for thermal physiology [90]. Acclimatization is an adaptive change that occurs when individuals are exposed to a natural climate, including geographical differences or seasonal changes [90]. Cold acclimatization is particularly relevant to indigenous populations that have lived in cold regions for a prolonged period, which generally have better cold tolerance. Acclimation is an adaptive change that occurs in response to experimental changes in particular climate factors, such as temperature, in a controlled environment [90]. Cold acclimation is achieved through repeated cold exposures in the laboratory, which generally increase human thermoregulation and cold tolerance. Habituation is a reduction in the physiological response or perception to a repeated stimulus [90].

There are several categorizations of cold adaptation based on the differences in thermoregulatory response observed in cold acclimatized populations compared to the non-acclimatized control groups [91–97]. It has been suggested that the duration and severity of cold exposure and systemic or local cold exposure during the adaptation period are the factors determining the types of cold adaptation [92, 93]. Here, we discuss several types of cold acclimatization and acclimation methods on the basis of the physiological response observed in populations that are regularly exposed to cold (cold acclimatization) and studies testing the effects of repeated cold exposure in the laboratory (cold acclimation).

In addition to the general types of cold adaptation, habituation of cold shock response after repeated cold immersions is summarized here. Moreover, cross-adaptation between cold and nonthermal factors, such as physical activity and hypoxia, in the thermoregulatory response to cold has been summarized, including recent studies conducted by our research group.

### Cold acclimatization

#### Habituation of metabolic response (shivering thermogenesis)

There have been several populations living in cold environments, such as indigenous hunter-gatherers in Central Australia (Indigenous Australians) or the Kalahari Desert (San people) and nomadic indigenous Norwegian people (Sami people), who have shown less metabolic heat production with habituation of shivering during sleep in cold environments [91, 98, 99]; population names have been replaced from the original manuscripts. In those indigenous populations, a greater reduction in their *T*_co_ while sleeping was observed than in the control group from a temperate climate [91, 98, 99]. This characteristic is called hypothermic acclimatization, which could be related to the lower metabolic heat production due to the habituation of shivering.

Habituation of shivering has been also observed in divers who regularly work in cold water. To assess the threshold temperature of shivering in laboratory experiments, the lowest *T*_w_ at which a participant can immerse for 3 h without shivering is defined as the critical water temperature (*T*_cw_) [100]. Studies have shown that the *T*_cw_ values of Korean cotton suit divers are lower than those of Korean non-divers [100, 101]. A lower *T*_cw_ implies a reduction in the *T*_w_ threshold to onset shivering. A similar response has been reported in several population groups, including Asians and Caucasians, engaging in diving work in Hawaii [102], with varying amounts of subcutaneous fat. Interestingly, after wet suits were used for several years, enabling divers to maintain their *T*_co_, the previously observed shift in the shivering threshold was diminished and became similar to that of non-divers [103]. Greek divers who engaged in sponge diving work with wetsuits also presented a shivering threshold similar to that of European non-divers [104]. This lack of adaptation of the shivering threshold observed in wetsuit divers suggested the necessity of repeated reduction in *T*_co_, and probably *T*_sk_, to obtain the habituation of metabolic responses to cold.

#### Metabolic acclimatization (non-shivering thermogenesis)

In an early study, a greater metabolic rate during sleeping in a warm environment was observed in Inuit people living in the Arctic region than in the control group [105]. The elevated BMR in the Inuit was considered as a type of metabolic acclimatization. More recent studies also reported a 3–19% greater BMR in indigenous circumpolar populations than in non-indigenous control groups [106]. Cotton suit Korean divers exhibit seasonal variation in their BMR, which is greater in winter and lower in summer [103, 107], whereas wet suit divers exhibit no seasonal variation [103]. The increase in BMR is probably due to the adaptation of divers to the cold water environment in winter.

In experimental animal studies, shivering thermogenesis is gradually replaced by a successive increase in NST during repeated cold exposure, and NA injection after the cold acclimation period increases BAT-derived thermogenesis more than that before acclimation [108]. In small mammals, adaptation to cold involves an increase in NST activated by NA [109, 110]. In human population studies, NA infusion significantly increased VO_2_ by 7.5% relative to the resting baseline in Korean cotton suit divers in winter but not in non-divers [111]. However, on the basis of the small increase in the metabolic response, they suggested that the development of NST was not the main feature of cold acclimatization in divers. In another population, NA infusion significantly increased NST by 15–17% relative to the baseline and plasma free fatty acid in indigenous people in Hokkaido (Ainu) but not in the control group living in Hokkaido [112]. They suggested the potential development of BAT in the indigenous population in the cold northern region of Japan, although it was not yet clear whether adult humans possessed active BAT at that time.

Recently, after the rediscovery of human BAT activity via the FDG-PET technique [4–6], metabolic acclimatization in human adults has become much clearer, with evidence of greater NST with higher BAT activity in winter [5, 24]. One clear example of the use of the FDG-PET/CT technique for determining population differences in BAT activity was the greater volume of active BAT in the Caucasian European group than in the South Asian group [20]. Recently, on the basis of the higher supraclavicular skin temperature, a surrogate measurement of BAT activity, indigenous Siberians (Yakut or Saha people) living in Yakutia presented greater BAT thermogenesis in mild cold environments than did the control population living near Chicago [113].

**Fig. 5:**
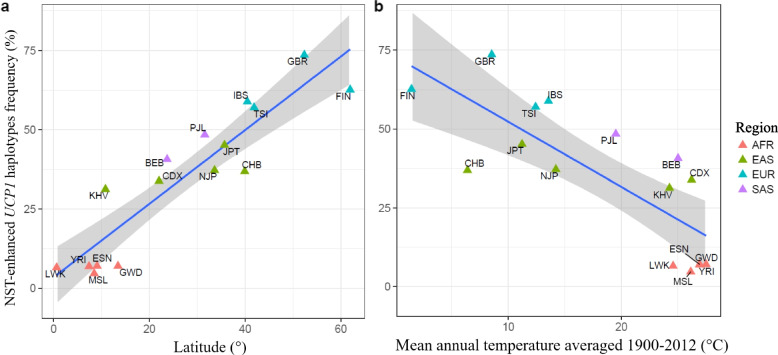
Correlations between the NST-enhanced haplotype and the latitudes (**a**) and mean annual temperatures (**b**) of global human populations deposited in the 1000 Genomes Project database The blue line and gray band represent a regression line and its 95% confidence interval, respectively. The colors of the triangles indicate the geographical regions of the populations, such as orange from Africa (AFR), green from East Asia (EAS), blue from Europe (EUR), and purple from South Asia (SAS). Modified from the original version in Nishimura et al. (2017). The abbreviations for each population group were found in the original article [122]

The uncoupling protein 1 (*UCP1*) gene is known as an obesity-related gene [114, 115], but the mechanism was unclear because BAT activity was not fully considered or ignored in adult humans at that time. However, as described above, BAT clearly exists even in adult humans and plays a role in thermogenesis and energy expenditure [5, 6]. Since UCP1 is known as a major thermogenic protein in BAT [116, 117], the relationship between the *UCP1* gene and obesity is important in the history of human adaptation and migration to cold regions. Recent genome-wide data in different human populations reported that some genes in northern Europeans and others, including *UCP1*, suggested experienced natural selection against cold climates [118, 119]. Another worldwide analysis has also suggested that positive selection acts on the *UCP1* gene with respect to cold adaptation [120]. In addition, age-related effects of the *UCP1* and *ADRB3* genotypes on BAT activity have been reported [121]. We focused on population differences in the UCP1 haplotypes and the enhancement of NST based on NST during mild cold exposure in a climate chamber and haplotype frequency in the 1000 Genomes Project database [122]. As shown in Fig. 5, the frequency of NST-enhanced UCP1 haplotypes was more frequent in populations from higher latitudes (Fig. 5a) and lower mean annual temperatures (Fig. 5b), i.e., the highest in Europeans, followed by Asian and African populations [122]. Other experimental studies reported a significant association between BAT activity assessed with FDG-PET/CT and the adrenoceptor beta 2 gene (*ADRB2*) genotype [123] and the leptin receptor gene (*LEPR*) genotype [124]. As described above, many genes related to energy metabolism or obesity have been identified, and such phenotypes might be linked to our past cold adaptation during the great journey of humans. However, few studies have assessed the direct relationship between genetic background and physiological function in humans, and further evidence is needed to clarify human evolutionary history and variation in adaptability to cold. These recent findings indicate that the activation of BAT is one of the major factors controlling metabolic cold acclimatization in humans, especially in enhanced NST during mild cold exposure. Additionally, genetic analysis suggested that natural selection controlled NST throughout the adaptive history of humans.

#### Insulative acclimatization

Early studies on indigenous Australians reported lower *T*_sk_, as well as habituation of shivering, during sleep in cold environments than did the control group [125]. Since peripheral cooling enables them to decrease heat loss from the skin, insulative acclimatization involves economical thermoregulation with the habituation of shivering and less heat production [98].

Studies have investigated the insulative cold acclimatization of Korean cotton suit divers. The level of vasoconstriction and tissue insulation (*I*_tissue_) calculated via the heat balance equation is assumed to be maximal at the end of 3 h of immersion in *T*_cw_ without shivering. Rennie et al. (1962) calculated the maximum *I*_tissue_ from the temperature gradient between *T*_co_ and *T*_cw_ and metabolic heat production in participants at steady state after 3 h of immersion in *T*_cw_. They reported a similar maximum *I*_tissue_ for Korean cotton suit divers and Korean non-divers measured in summer [100]. However, the maximum *I*_tissue_ for a given subcutaneous fat thickness measured in winter was found to be greater in divers than in non-divers [126], suggesting an adaptive increase in insulation due to the non-subcutaneous fat layer. Divers and non-divers showed similar blood flow in their limbs during immersion in 30–33 °C water, which is a typical range of *T*_cw_; however, the heat flux for a given blood flow tended to be lower in divers, suggesting that divers might adapt to changes in their countercurrent heat exchange in their limbs [127].

#### Population differences in CIVD

There are population differences in the CIVD response, with the African group having a smaller CIVD response and slower rewarming after hand immersion than those observed in the Caucasian group, whereas the Asian group shows an intermediate response [128]. This is thought to be one of the reasons for the high incidence of frostbite and NFCI in African populations [129].

There have been studies reporting greater CIVD responses in populations living in cold regions and working in cold environments, such as fishermen, divers, and cold storage workers. Norwegian Lapps and northern Norwegian fishermen presented significantly rapid CIVD onset compared with the control group, whereas no differences were detected in *T*_min_ and *T*_max_ [130]. Gaspe fishermen showed greater *T*_fing_, less finger pain, and lower blood pressure during hand cold immersion [131]. Compared with control workers and swimmers, ice chamber workers and swimmers presented higher* T*_mean_ and *T*_min_ values with shorter onset times [132]. A series of studies were conducted to evaluate the CIVD response of several populations living in Hokkaido, the northern island of Japan [133]. In general, Hokkaido natives, who were born and live in Hokkaido, presented greater CIVD responses with higher* T*_mean_ and *T*_min_ values and shorter onset times than did populations that moved from the main island of Japan [133]. Recently, it was reported that older women wetsuit divers (*haenyeo* in Korea and *ama* in Japan) living on Jeju Island in Korea presented higher *T*_min_ values than older non-divers [134], whereas a previous study reported that *T*_fing_ and blood flow were lower in cotton suit divers than in non-divers [135]. In addition, studies have reported a lower CIVD response in tropical indigenes than in temperate indigenes [136, 137]. On the basis of those findings in population studies, it is generally understood (or hypothesized) that the greater CIVD response in populations living or working in cold environments is due to cold acclimatization. However, in laboratory-controlled studies, findings on the effects of short-term repeated cold exposure on the CIVD response are inconsistent, and it is currently concluded that short-term acclimation in CIVD is unlikely to occur [53, 54, 97], which is explained later in the section on cold acclimation.

Population differences in CIVD response might in part depend on genetic variation. Recently, genetic analysis was conducted to assess whether the CIVD response in a Japanese cohort depends on genetic background (Yasukochi et al., 2023). According to genetic analysis, individuals without a CIVD response are differentiated from individuals with CIVD by genotypes of *COL4A2* (collagen type IV alpha 2 chain) and *PRLR* (prolactin receptor), which may affect blunted SkBF responses associated with endothelial NO-independent and neurogenic activity during finger cold immersion [65]. This result suggested that individual and population variations in the CIVD response might in part depend on genetic background.

### Cold acclimation

#### Habituation of metabolic response (shivering thermogenesis)

In laboratory experimental studies, it has been reported that metabolic heat production is reduced following repeated immersion in cold water [138–140]. A significant delay in shivering onset, measured in a protocol that gradually decreases *T*_w_, was observed after repeated cold water immersions [138]. The effect of the adaptation regime on the habituation of metabolic responses was also examined in their study, which compared repeated rest or exercising immersions in cold water [138]. The habituation of metabolic responses was significantly greater after repeated resting immersions, which induced greater decreases in *T*_co_ during the acclimation process than did repeated exercise immersions [138]. Tipton et al. (2013) confirmed that habituation to metabolic response is specific to the repeated reduction in *T*_co_ induced by cold water immersion during the habituation process. In their series of experiments, one experimental group repeated five times of 45-min immersions on average, resulting in 1.18 °C *T*_co_ reductions, and conducted testing immersion pre and post the acclimation process. A significantly blunted metabolic response was observed during post-test immersion until *T*_co_ decreased by 1.18 °C; however, no habituation was observed when *T*_co_ decreased further [141]. Budd et al. (1993) reported a significant reduction in the metabolic response and a delay in shivering onset, as measured by EMG, during 10 °C cold air exposure following 10 days of repeated cold water immersion, which lasted 30–60 min [139]. Similarly, a delay in shivering onset, accompanied by lower *T*_co_ thresholds, has been reported following repeated cold water immersions [140, 142], while there were individual variations in the thermoregulatory response obtained through cold acclimation, and some showed greater metabolic heat production [142], which might indicate an increase in NST.

As shown by the hypothermic acclimatization observed in cold-acclimatized populations sleeping in cold environments [91, 98, 99], a tendency toward a greater decrease in *T*_co_ during rest in cold water was observed after repeated cold water immersion, which might be associated with the habituation of the metabolic response [138]. Another research group reported a reduction in *T*_co_ during cold immersion after repeated cold immersions, with a significant reduction in the metabolic response [140]. In addition, a significant reduction in the resting *T*_co_ following an adaptation regimen has been reported by several investigators [140, 142]. However, several studies have not shown any evidence of hypothermic acclimation despite a recognized habituation of the metabolic response [139, 141]. This may be explained by an increase in vasoconstriction, as discussed in the section on insulative adaptation, and/or a reduction in shivering-induced heat loss may overcome the negative effect of decreased heat production. Recently, the habituation of the shivering response after repeated cold exposure was assessed via the EMG technique [26] and a subjective report [15], in addition to the increase in BAT activity, which is discussed in the next section.

#### Metabolic acclimation (non-shivering thermogenesis)

To compensate for the reduction in metabolic heat production observed during the habituation of the shivering response, an increase in NST (metabolic acclimation) after repeated cold exposure has been reported in animal studies [109, 110]. In a field experimental study in humans who stayed at Norwegian cold mountains for 6 weeks, an increase in the metabolic rate (metabolic acclimation) with higher *T*_sk_ (no insulative acclimation) was observed after the acclimation period [143].

The earliest laboratory-controlled study examined the metabolic response to NA infusion in a thermoneutral environment after 5 weeks of repeated cold exposure to 5 °C air for 8 h daily [144]. They reported a significant increase in VO_2_ after NA infusion in cold-adapted participants, which suggested metabolic acclimation with the enhancement of NST in humans [144]. In addition, as explained above, repeated cold water immersion led to habituation of the shivering response; at the same time, greater metabolic heat production was also observed [142], which might suggest that shivering was gradually replaced by NST. However, most of the laboratory studies that have examined cold adaptation after repeated cold water immersion have revealed no change in the metabolic response to NA injection [145], no adaptive change in the plasma NA concentration during cold water immersion [146], and no difference in the RMR under thermoneutral conditions [139]. Another study revealed a significant increase in plasma NA with lower *T*_sk_ (insulative acclimation) and lower *T*_co_ (hypothermic acclimation) during cold air exposure after repeated cold water immersion but no adaptive change in the metabolic response [147]. On the basis of these observations, it had been believed that there was no significant adaptation of NST in humans, probably because of the small amount of BAT in human adults compared with that in small mammals [148].

However, after the recent rediscovery of human BAT activity via the FDG-PET technique [4–6], the seasonal acclimation of BAT activity was shown to be greater in winter than in summer [5, 24]. In addition, it has been reported that experimental repeated short-term cold exposure enhances BAT activity in mild cold environments in healthy young people [9, 15, 25, 26], type 2 diabetic patients, and obese individuals [23, 29]. Some of these studies revealed evidence of metabolic acclimation with simultaneously elevated NST and BAT activity after cold acclimation [9, 15], whereas other studies did not find clear adaptation in NST. In addition to the increase in BAT activity after repeated cold exposure, habituation to shivering was simultaneously observed in terms of shivering intensity, as assessed via the EMG technique [26] and in a subjective report [15]. These findings suggest that an increase in BAT activity and NST (metabolic acclimation) would in part replace shivering thermogenesis (habituation of shivering) after cold acclimation in humans, but this finding is still not as clear as that in animal studies [108], probably because the amount of BAT in human adults is not enough to completely replace the ST, and the contributions of other thermo-effector tissues and the vasomotor response should be considered. Studies on individuals with type 2 diabetes and obesity reported that repeated cold exposure not only increases BAT activity but also increases glucose uptake into the SM, which is associated with increased translocation of glucose transporter type 4 (GLUT4) to the sarcolemma [23, 29]. We previously reported that forearm SM metabolism, assessed via near-infrared spectroscopy during low-intensity isometric handgripping, was enhanced after repeated local muscle cooling [149]. Additionally, animal studies have shown that repeated cold exposure leads to increased SM oxidative capacity in UCP1 knockout mice [150]. Another study reported an increase in sarcolipin-based thermogenesis in the SM of UCP1 knockout mice after repeated cold exposure [151]. These findings suggest a role for SM in the enhancement of NST following chronic cold exposure. The observed metabolic acclimation (enhanced NST), especially with evidence of BAT activation in human adults, represents significant progress in cold adaptation research.

#### Insulative acclimation

Several laboratory-based studies reported a significant decrease in *T*_sk_ during cold exposure after repeated cold water immersion [140, 142, 146, 147], which is a typical indicator of insulative acclimation, enabling a reduction in heat loss from the skin. O’Brien et al. (2000) examined the importance of repeated reductions in *T*_sk_ and *T*_co_ in determining cold acclimatization. They reported that repeated reduction in *T*_sk_ alone may be enough to induce insulative adaptation, reflected by increased total peripheral resistance (mean arterial pressure/cardiac output) during cold air exposure, even though no adaptive changes were observed in *T*_sk_ or *I*_tissue_, which are common indicators of insulative acclimation [152]. Additionally, we examined the effects of repeated mild cold immersion in 26 °C water, which caused only a modest increase (approximately 20%) in the metabolic response, on adaptation in the thermoregulatory response [153]. We found that this repeated mild cold immersion was sufficient to induce insulative acclimation, as indicated by a lower mean *T*_sk_ and lower SkBF assessed via laser Doppler flowmetry, but it did not lead to adaptive changes in the metabolic response. These observations suggested that insulative acclimation could be induced by skin cooling alone and relatively mild cold exposure, as long as the cold-induced vasoconstriction was repeated during the acclimation process.

#### No short-term acclimation in CIVD

As explained above, it is generally understood (or hypothesized) that the greater CIVD response in populations living or working in cold environments is due to cold acclimatization. However, in laboratory-controlled studies, findings on the effects of repeated cold exposure on the CIVD response are inconsistent and differ from the expectations of population studies. Some studies reported an enhanced CIVD response after short-term repeated cold exposure [154, 155]. On the other hand, many studies reported no change [156] or even blunted CIVD after short-term cold acclimation [152, 153, 157–160]. Considering the minor or negative effects of short-term cold acclimation on the CIVD response, several review articles have concluded that repeated cold exposure does not change the CIVD response [53, 54, 97]. Since repeated cold exposure could induce habituation to pain in the extremities and improve thermal comfort, even with no change (or decrease) in the skin temperature of the finger or toe [156, 159], the discrepancy between the subjective and physiological responses may increase the risk of cold injuries [156]. It is recommended that to prevent cold injuries, people should not rely on habituation through repeated cold exposure [53, 97].

#### Habituation of the cold shock response

It has been reported that the CSR is reduced following repeated immersion in cold water [67, 138, 141]. Recently, a systematic review and meta-analysis were conducted on previous studies on the habituation of CSR [71]. This review lists eligible studies on the habituation of CSR, summarizing research designs and major findings in the cardiovascular and ventilatory components of CSR. In addition, the mechanism of the CSR and habituation of the responses are discussed in the manuscript with citations of previous research, including animal studies [71]. Understanding the habituation process of the CSR has the potential to reduce the number of drownings through the clinical application of this process to high-risk groups.

The habituation of the cardiorespiratory CSR can be achieved following as few as several short-duration (2.5 to 3 min) cold water immersions [161–164]. It is strongly suggested that a repeated decrease in *T*_sk_ induces habituation in the CSR, and that this habituation occurs without a reduction in *T*_co_ [161, 162, 164, 165]. Six repeated 3-min head-outs immersed in 15 °C water were sufficient to significantly decrease the cardiorespiratory CSR in 10 °C water [162]. Even six repeated cold shower exposures induced habituation of the respiratory CSR during head-out immersion in 10 °C water [165], although habituation of the cardiac responses was not clearly observed. This may suggest that there are different mechanisms involved in producing habituation of the respiratory and cardiac CSR. Furthermore, repeated immersion on one side of the body could reduce cardiorespiratory CSR when the other side is immersed [161], indicating that habituation to CSR would occur centrally rather than peripherally. These observations indicated that repeated cold exposures with relatively small cold stimuli (higher *T*_w_ or smaller surface area exposed) can partly promote the habituation of the CSR to greater-intensity cold exposure. Additionally, in the case of a smaller cold stimulus, such as showers, the magnitude of habituation is determined by the rate of fall in *T*_sk_ and the body surface area exposed to the cold [165]. One study examined the permanence of the habituation of CSR. After six repeated short-duration cold water immersions, there was a gradual loss of habituation, but the habituation of hyperventilation and tachycardia was retained, in part, for up to 7 and 14 months, respectively [163].

As explained above, since repeated immersion of one side of the body induces habituation to CSR when the other side of the body is immersed in cold water, habituation to CSR occurs centrally rather than peripherally [161]. In an animal study, no change in the static and dynamic characteristics of cold receptors in the nasal area of cats was reported after 2 months of cold exposure to 5 °C air [166]. However, after 4.7 years of long-term continuous cold exposure to 5 °C air, a change in the dynamic activity of cold receptors was observed in cats [167]. This is supported by the evidence of a significantly smaller number of cold spots observed in long-term cold-adapted humans, who have lived in polar regions for at least 5 years and have been working outdoors [168]. Compared with these studies, the duration for which the habituation of CSR is achieved is far too short to induce adaptive changes in cold receptors. Thus, the habituation of CSR is primarily mediated by central phenomena rather than peripheral changes.

An animal study in cats suggested that reflex respiratory responses to cold are mediated at the midbrain, and that the cerebrum is not essential for acute responses [169]. However, the involvement of the frontal area of the cerebral cortex in the habituation process has been reported [170, 171]. It has been suggested that the frontal cortex is important in facilitating the habituation of cardiovascular responses but not in maintaining established habituation [170]. A study in leucotomized patients whose frontal cortex had been removed indicated the involvement of the frontal cortex in the habituation process of cardiovascular CSR in humans [171]. A more recent study reported that the cerebral cortex plays a critical role in top-down inhibitory control of autonomic centers located in the brainstem during innocuous whole-body cooling [172]. The precise location and nature of the central changes responsible for the habituation of CSR are still under discussion. More information on the mechanism of the habituation of CSR is summarized in our recent review article [71].

#### Cross-adaptation with exercise training

##### Cross-adaptation

The concept of cross-adaptation is explained as follows: “long-term exposure to a given environment not only leads to increased tolerance to the particular environment but also leads to gain (positive cross-adaptation) or loss (negative cross-adaptation) tolerance to other factors” [173]. Although the concept and studies of cross-adaptation have been known and conducted for many years [173–175], studies on cross-adaptation in humans remain rare [176]. Here, we focus on cross-adaptation between cold and nonthermal factors, especially the effects of repeated stimulus exercise and hypoxia on the adaptive thermoregulatory response in cold environments.

##### Exercise training effect on thermoregulation in cold

Studies have investigated the effects of exercise training or physical fitness level on thermoregulation in cold environments. The maximal oxygen uptake (VO_2max_), which is regarded as a parameter of aerobic capacity and physical fitness, is positively correlated with metabolic heat production, mean *T*_sk_ during cold exposure, and skin thermal conductance (dry heat loss divided by the *T*_co_ to *T*_sk_ gradient) at the end of cold exposure [177, 178]. The higher metabolic heat production observed in the trained individuals led to better cold tolerance, whereas the higher *T*_sk_ during cold exposure promoted more heat loss from the skin. In both studies cited above, VO_2max_ was negatively correlated with % body fat, which might be a reason for the lower* T*_sk_ in untrained individuals, since a thicker subcutaneous fat layer in the trunk region results in a lower *T*_sk_ above it [179, 180]. Indeed, % body fat was negatively correlated with mean *T*_sk_ and skin thermal conductance [177, 178]. In this context, additional analysis revealed that the slope of the mean* T*_sk_ as a function of % body fat was significantly steeper in the trained group than in the untrained group, which suggested greater peripheral vasoconstriction in trained individuals when the effect of the % body fat was adjusted [178]. The greater vasoconstriction in trained individuals was supported by a study reporting an inverse association between VO_2max_ and a change in *T*_fing_, which reflects the skin vasomotor response well, with a minimum effect of subcutaneous fat [181], although no correlation was observed between VO_2max_ and forearm SkBF measured via laser Doppler flowmetry. In addition, the stronger cold-induced vasoconstriction in fingers in physically fit individuals with greater VO_2max_ was confirmed by measuring changes in the diameter of the finger blood vessels via a near-infrared transmission imaging technique during 90 min of cold exposure to 10 °C air [182]. Similarly, greater vasoconstriction measured via laser Doppler flowmetry and lower *T*_sk_ was observed in the upper extremities of trained individuals than in those of untrained individuals during mild cold exposure [183]. These greater vasoconstrictor responses would lead to better cold tolerance by increasing the insulation.

As explained above, there was a positive correlation between VO_2max_ and CIT [177, 178], suggesting that individuals with higher fitness levels might have better cold tolerance in terms of increased metabolic heat production. On the other hand, one study reported a lower CIT during 90 min of cold exposure at 10 °C in individuals with higher fitness levels [181]. The lower CIT could be explained by the greater BMR observed in individuals with higher VO_2max_. It was suggested that the greater BMR enabled them to maintain their *T*_co_ with a less adaptive metabolic response [181]. Repeated exercise training increases the mitochondrial volume density in skeletal muscle [184, 185], which could lead to greater BMR, NST, and ST, although there could be some complementary response between the BMR and CIT [181]. In addition, there could be complementary thermogenesis between NST and ST, as shown in our study, which reported that lower *T*_co_ in individuals with less BAT and NST induced an earlier onset of shivering in the SM [8]. Although it depends on the strength of cold exposure and the interaction between vasomotor and metabolic responses to different thermo-effectors, exercise training could enhance metabolic and vasoconstriction functions, which would contribute to maintaining *T*_co_ more efficiently.

Recently, the effects of exercise training on human BAT activity have been investigated in several studies and summarized in review articles [186, 187]. Here, we introduce previous findings on exercise training and human BAT activity, with some interpretations of the potential mechanism from the perspective of thermal physiology.

BAT activity assessed by FDG-PET/CT during mild cold exposure was reported to be significantly lower in endurance-trained athletes than in untrained sedentary athletes [183]. Similarly, a lower tendency in cold-induced BAT activity was observed in athletes, with a greater trend in the RMR [188]. Although their results did not reveal a correlation between RMR and BAT activity [188], some studies reported a negative correlation between BMR and CIT [181, 189]. The greater BMR in trained individuals could be due to inevitable mitochondrial thermogenesis in the SM [190]. In addition, Vosselman et al. (2015) reported that athletes showed greater vasoconstriction in their arms and lower *T*_sk_ during mild cold exposure [183], which might enable them to induce less NST and BAT activity to maintain *T*_co_. Thus, the lower level of cold-induced BAT activity in the athletes might be explained in part by their greater BMR and/or greater cold-induced vasoconstriction.

A previous study investigated the effects of high-intensity interval training and moderate-intensity continuous training on insulin-stimulated BAT activity, which did not include the effects of cold-induced thermoregulation [191]. The insulin-stimulated FDG uptake in the BAT was significantly decreased after a 2-week training session in the group with higher BAT activity at preintervention, whereas FDG uptake in the quadriceps femoris was significantly increased [191]. Similar to the findings of less cold-induced BAT activity in athletes, as explained above [183, 188], this result also indicated that physical activity might downregulate BAT activity, even when it was assessed without a cold stimulus. This approach might constitute a reasonable biological strategy for reducing energy expenditure in BAT to compensate for the greater energy expenditure in SM with physical activity. In addition, because of the greater degree of inevitable thermogenesis in the SM [190], there may be less need for BAT to generate additional heat.

On the other hand, some studies have reported a positive effect of exercise on human BAT activity. There was a significant positive correlation between the volume of habitual physical activity (weekly metabolic equivalents) and BAT activity assessed via FDG-PET/CT without cold exposure [192]. In addition, significantly greater BAT activity was observed in groups with high-intensity levels of habitual physical activity than in those with low- or moderate-intensity exercise habit [192]. Compared with moderate-intensity endurance exercise, vigorous-intensity exercise, such as resistance exercise, activates the sympathetic nervous system [193, 194], which is known as the major pathway for activating BAT. Considering the potential effect of high-intensity exercise training on BAT activity through repeated activation of the sympathetic nervous system, several studies have been conducted. It has been reported that people with vigorous-intensity physical activity habits have significantly greater BAT vascular density in the supraclavicular region [195], which was assessed by time-resolved near-infrared spectroscopy without cold stimulus [196, 197]. On the other hand, in their longitudinal training intervention study, no significant change in BAT vascular density was observed in either the group with 10 weeks of high-intensity resistance training or the control group without training [198]. However, the change in BAT density was positively correlated with the strength training volume (weight × repetitions × sets) and the frequency of mild cold exposure during training (the number of occasions when the training room temperature was less than the median value) [198]. Thus, repeated stimulation of the sympathetic nervous system induced by high-intensity exercise, especially cold exposure, might increase BAT activity. Further investigations are needed to clarify the effect of exercise on human BAT activity from the viewpoint of exercise intensity and environmental conditions, with some evidence of activity in the sympathetic nervous system and body temperature regulation during training interventions. In addition, the methodological variation in assessing BAT activity with/without cold stimulus could be one of the major reasons for the discrepancy among the studies reporting positive [192, 195] and negative [183, 188] or no effect [199] of exercise training on BAT activity.

##### Exercise training in cold

As indicated above, exercise training itself might enhance or impair cold tolerance depending on the effectors that induce thermoregulatory responses. It has been suggested that repeated exercise in cold environments might increase cold tolerance [198]. Although the cross-adaptation between exercise and cold has been studied for many years [175, 200], the interactive effects of exercise and cold stimuli have not been well characterized. Cold acclimatization in divers has been well studied, as explained above in this manuscript and in several review articles [201–203]. Indeed, adaptation in their thermoregulatory response could include cross-adaptation with exercise during their diving work.

Habituation of shivering has often been observed in Korean women divers, as shown by the elevation of the shivering threshold *T*_w_ compared with non-divers [100, 101]. A similar response has been reported in other populations, including Asians and Caucasians, engaging in diving work [102]. Moreover, metabolic acclimatization has been observed in cotton suit divers with greater BMRs in winter [103, 107]. In addition, NA infusion significantly increased VO_2_ by 7.5% relative to the resting baseline in cotton suit divers in the winter [111]. Although the development of NST was not the main feature of cold acclimatization due to the small increase in the metabolic response, it might be interpreted differently today after the rediscovery of human BAT. There might be a replacement of cold-induced shivering with greater BMR and/or NST in cold-adapted divers. There have been studies reporting insulative cold acclimatization in divers. The maximum *I*_tissue_, calculated from the *T*_co_ to *T*_cw_ gradient and metabolic heat production, for a given subcutaneous fat thickness was greater in divers than in non-divers in winter [126], suggesting greater insulation due to the non-subcutaneous fat layer. Although divers and non-divers showed similar blood flow in their limbs during immersion in 30–33 °C water, the heat flux for a given blood flow tended to be lower in divers, suggesting that there might be an adaptive change in their countercurrent heat exchange in their limbs [127].

In addition to the general thermoregulatory adaptations of divers, few studies have focused on adaptative changes in the level of skeletal muscle (fiber type, capillarization, and metabolism). Korean divers who regularly work in cold water (10–12 °C in winter and 25–27 °C in summer) have a higher percentage of type IIx (fast glycolytic) fibers and a lower proportion of type IIa (fast oxidative glycolytic) fibers in the vastus lateralis than physically active controls do [204], which might be due to the repeated recruitment of fast-twitch fibers in cold [205–207]. The recruitment of fast-twitch glycolytic fibers has been supported by evidence from human studies reporting greater anaerobic metabolism during exercise in cold [208–210]. We also reported that forearm muscle oxygen consumption during handgrip exercise, which was estimated by the oxy hemoglobin and myoglobin (Oxy [Hb + Mb]) curve during arterial occlusion, decreased with decreasing muscle temperature [149]. The suppression of aerobic metabolism in hypothermic SM might compensatory increase the contribution of anaerobic metabolism to maintaining the same exercise intensity as that in normothermic SM. In a cold adaptation study in animals, a shift in fiber type from type I to type IIa fibers in the rat soleus muscle was observed after repeated cold exposure [211]. In addition to the specific muscle fiber type composition, Korean breath-hold divers had greater capillary density and capillary-to-fiber ratios than physically active controls did [204]. Similarly, in animal studies, chronic cold exposure has been reported to increase capillary density and/or the capillary-to-fiber ratio [212–214].

However, in a study of Indonesian breath-hold divers working in moderate water (29–30 °C), no difference in muscle fiber type composition or capillarization was observed between divers and age-matched non-divers (Park et al., 2005), which differed from the results observed in their study of Korean divers working in cold water [204]. The discrepancy between these studies could indicate that the greater proportion of fast glycolytic fibers and capillarization in the SM in divers working in cold water [204] might be induced by the cold stimulus itself and/or the lower O_2_ supply to the working muscle due to cold-induced vasoconstriction [215–217] rather than hypoxia due to beath-holding. On the basis of these findings and the potential mechanisms of the specific fiber type and capillarization of SM in cold-adapted divers, we conducted a training intervention study to assess the effects of moderate-intensity exercise training in cold water on adaptations in the metabolic response, including SM oxygenation, anaerobic metabolism, and exercise performance [1, 218].

The participants in our intervention study were divided into cooling and thermoneutral control groups, which were subjected to 4 weeks of exercise training (3 times a week, 12 times in total) with their lower bodies immersed in cold water (15 °C) and thermoneutral water (33 °C), respectively [1, 218]. In a training session, each cooling and thermoneutral group was subjected to 30 min of exercise in cold and thermoneutral water following 30 min of resting immersion at each temperature, respectively, at a lactate threshold (LT) workload predetermined in thermoneutral water. This protocol could induce a greater contribution of anaerobic glycolytic metabolism, as shown by the higher blood lactate (La) concentration in the cooling group [1, 210, 218].

**Fig. 6:**
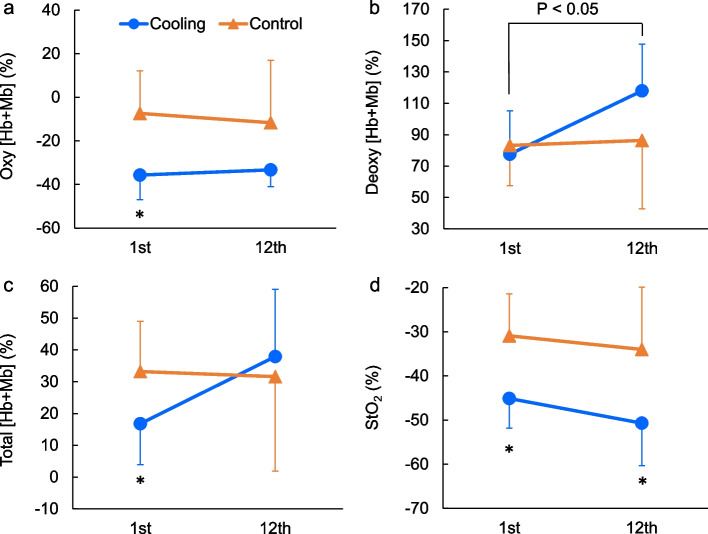
Overall phase average values of percent change relative to the resting baseline in Oxy [Hb + Mb] (**a**), Deoxy [Hb + Mb] (**b**), Total [Hb + Mb] (**c**), and StO_2_ (**d**) in the vastus lateralis during the 30-min submaximal cycling exercise at the 1st and 12th training sessions in the cooling and control groups The values are the means±SDs. *Significant difference between groups at each training session. The figures were drawn on the basis of the data in the original article [218]

As a result, a significant increase in muscle deoxygenation during submaximal exercise was observed after the intervention of the training in cold water [1, 218]. The deoxyhemoglobin and myoglobin (Deoxy [Hb + Mb]) in the vastus lateralis during the LT intensity exercise were significantly greater in the final (12th) training session than in the first training session in the cooling group (Fig. 6b), whereas no training effect was observed in Oxy [Hb + Mb] (Fig. 6a). The increase in muscle deoxygenation might indicate an improvement in muscle oxygen extraction. In acute response to hypothermic SM, greater muscle Deoxy [Hb + Mb] [209], less muscle Oxy [Hb + Mb] [210], and less tissue oxygen saturation (StO_2_) [209, 210] were observed in our previous reports, indicating a lower O_2_ supply to the working muscle due to cold-induced vasoconstriction relative to O_2_ consumption in SM. The lower StO_2_ in cold water was confirmed at the first and final intervention sessions (Fig. 6d), which indicated that the hypoxic condition in the working muscle was repeated during the intervention. When the working SM was repeatedly subjected to hypoxia, there was some adaptation in the microvascular network, such as an increased capillary-to-muscle fiber ratio [219, 220], which has been reported as a training effect of hypoxic training. As explained above, breath-holding divers working in cold water had greater capillary density and capillary-to-fiber ratios than the controls did [204], which was similar to the greater capillarization observed in cold-adapted animals [212–214]. In our study, total hemoglobin and myoglobin (Total [Hb + Mb]) during the first training session were significantly lower in the cooling group than in the control group (*P* < 0.05), whereas no group difference was observed at the final session (Fig. 6c), which might indicate relatively enhanced muscle perfusion after training in cold water, potentially due to microvascular remodeling [204, 212–214]. Considering these findings, repeated exercise in cold environments with a limited O_2_ supply to the working muscle might improve muscle O_2_ extraction through microvascular remodeling. Interestingly, a similar training effect on muscle deoxygenation [221, 222] and enhanced muscle perfusion [222] was observed after the intervention of hypoxic training. There could be a similarity between exercise training in a hypoxic environment and that with hypothermic SM at the point of O_2_ restriction at the working muscle level, as shown by the lower StO_2_ in the vastus lateralis throughout the training sessions in the cooling group (Fig. 6d).

Before and after the training period, the participants conducted performance tests for assessing the LT workload in thermoneutral water, VO_2max_ using a bicycle ergometer on land, and a Wingate test for evaluating anaerobic power. As a result, no training effect on aerobic metabolism was observed for the LT level or VO_2max_ in either group. However, training in cold water, even at moderate workloads, significantly improved anaerobic power [1, 218]. In the Wingate test, greater average power was observed after the training period than before the intervention only in the cooling group. Additionally, the pre to post percent change in the time to peak power was significantly shorten in cooling group compared to the thermoneutral control group (− 11.5 ± 17.6% and 15.6 ± 28.0% in the cooling and control groups, respectively). These results indicated an improvement in anaerobic metabolism and a greater contribution of fast-twitch fibers after training intervention in cold water.

On the basis of these findings, we propose a new training method, “hypothermic skeletal muscle training,” which utilizes the physiological strain under cold conditions as an additional physiological load [1]. This training can provide a hypoxic-like environment at the SM level due to cold-induced vasoconstriction [215–217] and a leftward shift in the oxyhemoglobin dissociation curve at lower temperatures [223, 224]. In addition to the lower O_2_ supply and O_2_ utilization in the working muscle, neuromuscular functions are also impaired in cold. A lower shift in EMG frequency with lower muscle temperature has been consistently reported during exercise in cold [225–229], which is associated with a reduction in nerve conduction velocity [230] and muscle conduction velocity [225, 228]. To compensate for the decline in neuromuscular function, a greater number of motor units are recruited in cold to maintain the same workload under thermoneutral conditions [231], which could include higher threshold fast-twitch fibers [206, 207]. On the basis of these physiological mechanisms, hypothermic SM training in cold water, even at moderate workloads, can repeatedly promote glycolytic metabolism [209, 210] and improve anaerobic power [1, 218], which could support the evidence that greater glycolytic muscle fiber components are observed in cold-adapted divers [204].

#### Cross-adaptation with hypoxia

It is well known that some ethnic groups, such as the Andean or Tibetan people, have adapted to high-altitude environments. At high altitudes, many comprehensive factors affect human physiological functions; in particular, a hypobaric environment leads to hypoxia in the human body, and the secondary air temperature decreases with increasing altitude. Therefore, there is a possibility of cross-adaptation to hypobaric hypoxia and cold environments at high altitudes.

Little and Hanna reviewed and summarized the thermoregulatory response during whole-body and distal local cold exposure in various highlanders from the 1960s to the 1970s [232]. Compared with lowlanders, most highlanders, including Andean, Sherpa, and Ladakh, as well as even sojourners at high altitudes, presented elevated BMRs [232]. Similarly, Tibetan nomads were reported to have higher BMRs than the BMRs predicted via WHO equations without any seasonal differences [233]. These findings of higher BMR in highlanders could contribute to their greater cold tolerance. The transient increase in the BMR among sojourners seems directly linked to altitude-associated hypoxia, whereas the chronically elevated BMR of high-altitude natives might be attributed to the combined effects of hypobaric hypoxia and cold [232]. Interestingly, Ladakh highlanders induced much less shivering (habituation of shivering) than did lowlanders, with a similar increase in VO_2_ during 10 °C cold air exposure at sea level [234]. This might be due to their greater BMR and/or greater increase in NST, although there was a lack of supporting data. However, the upregulation of NST, which might include BAT as a thermo-effector, would be advantageous in cold environments but not preferable in hypoxic environments, as it leads to increased oxygen demand and energy expenditure. In fact, a study on mice revealed that BAT mass increased more with simple cold acclimation than when they were exposed to a combination of cold and hypobaric hypoxia [235]. Additionally, the metabolic response to NA infusion increases after cold acclimation, but there is no adaptive change after a combination of cold and hypobaric hypoxia, and the response decreases after chronic hypoxia [235]. However, further studies are needed to investigate NST, including BAT activity, in highlanders.

With respect to the vasomotor response, a greater *T*_sk_ of the extremities was often observed in highlander populations than in lowlanders during whole-body cold air exposure ranging from 0 to 10 °C [232], suggesting greater blood flow to the extremities in moderately cold environments. In general, chronic hypoxic exposure induces angiogenesis and an increase in hemoglobin, which is mainly mediated by hypoxia-inducible factors (HIFs) [236]. Increased angiogenesis might be one of the factors that induces increased SkBF and *T*_sk_ in populations at high altitudes.

It has been reported that Tibetans living at high altitudes have greater blood flow due to higher NO levels than do those living at low altitudes, while the hemoglobin concentrations are similar [237]. This is one of the key adaptations at high altitudes to increase oxygen delivery, which collaterally increases blood circulation to the extremities and may improve peripheral cold tolerance, as reflected in increased *T*_sk_ [232]. However, in Andeans who live at similar high altitudes, there is no increase in the amount of blood flow; instead, they have higher hemoglobin concentrations to increase oxygen delivery, which results in increased blood viscosity [238]. In our recent study, a higher peripheral *T*_sk_ of Tibetans in Nepal (33.4 °C) [239] was observed than that of Andeans in Bolivia (32.8 °C) [240], despite both populations living at similar high altitudes. These findings suggest that highland people have greater blood flow under cold conditions, especially Tibetan people, who might have more flexible vasomotor functions and greater local cold tolerance due to their unique adaptation to high altitudes.

Mathew et al. reported that Ladakh highlanders presented better local cold tolerance in the CIVD response, with higher *T*_fing_ during hand cold immersion than did lowlanders tested both at 3500 m altitude [241] and at sea level [234]. Similarly, during severe local foot cold exposure in cold water, Andean highlanders presented higher *T*_sk_ values on their toes than did lowlanders from a US university [242]. Furthermore, compared with male Andeans, female Andean highlanders presented a greater CIVD response during hand cold immersion, reactive hyperemia, and recovery in their *T*_fing_, with the lowest response observed in lowlanders from the USA who had resided at high altitude for at least 1 month [243]. In contrast, Tibetan highlanders presented a lower mean *T*_fing_ during finger cold immersion than Japanese lowlanders did at 2260-m altitude [244]. This might indicate a blunted CIVD response or strong vasoconstriction in Tibetans, although it was suggested that the lower mean *T*_fing_ in Tibetans might be due to their lower baseline *T*_fing_ [244].

In a laboratory study conducted in a climatic chamber with lowland participants, higher *T*_fing_ during a finger cold immersion test was observed under simulated 4000-m conditions than under 2000-m conditions [244]. On the other hand, another study with lowlanders reported that acute exposure to mild hypoxia (*FiO*_*2*_ = 0.17) did not change the parameters of the CIVD response in *T*_fing_ during finger cold immersion compared with normoxic conditions [245]. Similarly, compared with normoxia, acute exposure to normobaric hypoxia (*FiO*_*2*_ = 0.14) did not affect the cold-induced decrease in *T*_fing_ during hand cold immersion; moreover, rewarming of *T*_fing_ was markedly impaired in hypoxia [246]. On the basis of these findings, there is a lack of consensus on the CIVD response during acute hypoxic exposure in lowlanders.

One study investigated how lowlanders adapt to hypobaric hypoxia when moving to high altitudes and staying there. A study revealed that lowlanders presented a markedly impaired CIVD response at 3500 m compared with their response at sea level [241]. This diminished response persisted throughout the 3-week stay, although certain parameters were at their worst during the second week. In addition, after residing at high altitude for 1 year, lowlanders presented an improved CIVD response compared with their initial 3-week acclimation, although their response remained weaker than that of native highlanders [241]. Since those findings include both effects of repeated exposure to hypoxia and cold, it is difficult to conclude that hypoxia might impair the CIVD response in lowlanders.

The duration of stay at high altitude could be a remarkable factor in determining the CIVD response, although methodological differences among the studies also influence the results. Acute, repeated, or chronic hypoxia can lead to different CIVD responses, and population differences also exist even among native highlanders. While hypoxic conditions can alter the CIVD response, the underlying mechanisms remain unclear. Further studies, both in the laboratory and in the field, are needed to resolve this outstanding issue.

### Factors affecting cold adaptation type

The variability of cold adaptation has been categorized in several articles, and the possible factors affecting cold adaptation have been discussed [91, 94–97, 247]. Originally, Hammel (1964) categorized metabolic, hypothermic, and insulative-hypothermic acclimatization on the basis of studies of indigenous populations in different climatic regions.

LeBlanc (1975) categorized two different types of cold adaptation in humans produced by continuous moderate or intermittent severe cold exposure on the basis of findings from animal studies and human studies [247]. In animal studies, continuous moderate cold exposure with shivering led to metabolic adaptation, replacing shivering with increased NST and increasing both metabolic sensitivity to NA [248] and NA excretion to cold [249]. In contrast, intermittent severe cold exposure, involving short-duration exposure to colder temperatures on local skin areas without significant shivering, did not increase metabolic sensitivity to NA [110]. In humans, continuous moderate cold exposure, as observed in Australian indigenes [91, 98], led to habituation to shivering, similar to the findings of animal studies, whereas at that time it was not considered to be replaced with enhanced NST [247], although some studies had already reported greater metabolic sensitivity to NA in cold-adapted populations [112]. Inuit and Gaspe fishermen, categorized as experiencing intermittent severe cold exposure due to clothing that only exposes their extremities and face, shivered as many [250] or even more than the control groups did [251]. In addition, Gaspe fishermen presented a relatively high mean *T*_sk_ during whole-body cold exposure [251], and both Gaspe fishermen and Inuit presented relatively higher *T*_fing_ and a relatively small increase in blood pressure during hand cold immersion [131, 247]. These findings suggest that intermittent severe cold exposure in humans leads to habituation in cardiovascular responses.

Bittel (1987) categorized metabolic and insulative types of cold acclimation on the basis of individual thermoregulatory responses to cold air exposure obtained after repeated cold water immersions [142]. He defined metabolic acclimation as a greater metabolic response after cold adaptation and insulative acclimation as a lower *T*_sk_. From observations of individual case data, the possible effects of morphological characteristics and physical fitness on adaptation type have been described [142], although they have not been clearly investigated. The effect of morphological characteristics on adaptation type may be due to an indirect consequence of differences in repeated *T*_co_ reduction during the adaptation process, which is associated with physical characteristics.

It has been reported that the habituation of the metabolic response to cold is significantly greater following repeated resting immersions in 15 °C water, which causes a greater decrease in *T*_co_ than does repeated immersion with exercise [138]. Another research group also examined the relative importance of repeated reductions in *T*_sk_ and *T*_co_ in determining the type of cold acclimation [152]. They conducted repeated cold water immersion in two groups: one with exercise, which induced cooling in *T*_sk_ alone, and one without exercise, which induced cooling in both *T*_sk_ and *T*_co_. They reported that repeated reduction in *T*_sk_ alone may be enough to induce an insulative adaptation, reflected by increased total peripheral resistance during cold air exposure, even though no adaptive changes were observed in *T*_sk_ or *I*_tissue_. In contrast, a repeated reduction in *T*_co_ was necessary for NA excretion, while neither group showed any adaptation in terms of metabolic response during cold air exposure [152].

In our subsequent study, we focused on the habituation of the metabolic and ventilatory response specific to the repeated reductions in *T*_sk_ and *T*_co_ during the cold acclimation process [141]. Here, one group was subjected to repeated 5-min cold immersions in 12 °C water, which induced cooling in *T*_sk_ alone, whereas another group was subjected to prolonged cold immersions, which reduced both *T*_sk_ and *T*_co_, with *T*_co_ decreasing by 1.18 °C. We found that repeated skin cooling in 12 °C water alone led to habituation of the CSR, although this was insufficient to induce habituation in the metabolic response during longer immersion periods with *T*_co_ reduction [141]. However, repeated *T*_co_ reduction led to significant habituation of the metabolic response, which was specific to *T*_co_ levels repeatedly experienced during the acclimation process; however, it reverted to an unhabituated response when *T*_co_ fell below these previously experienced levels [141]. These observations suggest that habituation of the metabolic response to cold may depend on the magnitude of the cooling repeatedly experienced. Previously, hypothermic adaptation was thought to result from habituation in the metabolic response to cold [98, 140], although our study showed that an unhabituated metabolic response may return when *T*_co_ drops below the levels experienced during habituation development [141]. Additionally, we examined the effects of repeated mild cold immersion in 26 °C water, which caused only a modest increase (approximately 20%) in the metabolic response, on adaptive changes in thermoregulation [153]. We found that this repeated mild cold immersion was sufficient to induce an insulative adaptation, as indicated by a lower SkBF and mean *T*_sk_, but it did not lead to adaptive changes in the metabolic response. These findings suggest that the type of cold adaptation could depend on the specific thermoregulatory responses repeatedly experienced during the adaptation process.

On the basis of more recent studies, we update the potential factors affecting the type of cold adaptation, with a particular focus on findings related to human BAT activity. Unlike other thermo-effectors, such as SM or white adipose tissue, human BAT has considerable individual variations in its activity and contribution to the CIT. As a result, the type of cold adaptation experienced may be influenced by both the preadaptation state and the adaptation potential of an individual’s BAT activity. For example, in young healthy individuals with high preadaptation BAT activity, repeated cold exposure has been shown to induce metabolic acclimation with increased NST and BAT activity, whereas no adaptive changes in the metabolic parameters in the SM have been observed [15, 25]. On the other hand, in type 2 diabetic patients and obese individuals with low preadaptation BAT activity, repeated cold exposure not only enhances BAT activity but also increases glucose uptake into the SM [23, 29]. Considering these findings, the contributions of other thermo-effectors to adaptive changes may differ depending on an individual’s preadaptation state and adaptation potential in BAT activity.

Finally, studies on cross-adaptation between cold and nonthermal factors, especially physical activity during the cold adaptation process and the fitness level in the preadaptation state, could affect adaptation in the thermoregulatory response [200].

The factors affecting cold adaptation types are quite complex, and findings are variable, making it difficult to summarize them in a simple flow chart. However, as mentioned above, they are likely determined by the combination of repeated body temperature changes and/or thermoregulatory responses during the adaptation period, as well as the preadaptation states of various thermo-effectors, individual physiological characteristics, and nonthermal factors that may influence these responses.

## Practical implications

Based on the findings on human cold adaptation summarized in this review, authors suggest several practical implications, as follows.

“Living in mild cold” or adopting a lifestyle that regularly exposes one to cold, such as walking in cold air, can enhance BAT activity and increase NST, leading to improved cold tolerance. This may also benefit metabolic health, such as reducing obesity and diabetes, by increasing energy expenditure. However, caution is needed regarding potential side effects, such as an increased risk of cardiac events, especially in the elderly, who tend to show a greater rise in blood pressure in response to cold [252]. Considering the side effects of cold exposure and thermal comfort, food intake or skin application of chemicals that activate transient receptor potential channels, such as capsaicin, catechin, and menthol, has been proposed as a promising method to activate human BAT [16, 253]. These approaches also serve as nonthermal factors for enhancing the thermoregulatory response and cold tolerance.

Regarding the CIVD response, while populations living or working in cold environments show a greater CIVD, recent laboratory studies suggest “no short-term acclimation in CIVD” [53, 97]. Therefore, individuals such as outside workers or athletes in cold environment should not rely on habituation to prevent cold injuries in their distal extremities.

“Habituation of the CSR” after repeated cold water immersion could help reduce the risk of drownings during the initial phase of accidental cold immersion [71]. This process could be clinically applied to high-risk groups, such as coast guards, fishermen, and others working near cold water. Additionally, the CSR habituation protocol could be applied to triathletes and open water swimmers to reduce the negative effects of CSR on their swimming performance when rushing into cold water.

“Training in cold” can benefit a wide range of individuals, from sedentary people to outdoor workers, by improving the general thermoregulatory response to cold. We propose a new training method, “hypothermic skeletal muscle training,” which utilizes the physiological strain under cold conditions as an additional load [1, 218]. This training can enhance anaerobic power, even with moderate mechanical intensity exercises repeated during the session, making it a potential new training method for athletes. To prevent injuries to hypothermic skeletal muscle, exercise intensity should be low to moderate. Simple exercises, such as cycling on an ergometer or treadmill running at a steady pace, are recommended.

Finally, there are individual variations in thermal sensation and comfort, even in indoor working environments for sedentary workers [254]. In addition to the gender and morphological difference, cold adaptation can contribute to these variations. Adjusting the working thermal environment according to individuals’ adaptation levels will be recommended in future offices, especially from the perspectives of diversity, inclusion, and globalization.

## Conclusions

In this review, we first provided an overview of human thermoregulatory responses in cold environments, with recent updates, particularly on the role of human BAT. Along with general thermoregulation, we covered the CIVD response during local cold immersion of the extremities and the cold shock response upon immersion in cold water, with recent insights into their physiological mechanisms. Next, the main topics of this review regarding cold adaptation, categorization of cold acclimatization/acclimation into habituation of shivering, and metabolic and insulative adaptation were provided, with some recent updates. Especially, the rediscovery of human BAT has clarified findings of metabolic acclimation, where increased NST and BAT activity replace shivering, which was not well understood before. Furthermore, cross-adaptation between cold and physical activity was reviewed, emphasizing findings of the effects of physical activity on the thermoregulation in cold. Especially, the effects of exercise training in cold were summarized including recent intervention study investigating the effect of training in cold water on skeletal muscle metabolism. We found that the hypothermic skeletal muscle training increased muscle deoxygenation during submaximal exercise and improved anerobic power in the Wingate test. The effects of hypoxic environment on cold adaptation were also summarized based on studies on highland populations and laboratory research. Elevated BMR and higher distal skin temperature observed in highlanders could contribute their higher cold tolerance. Referring to the above, comprehensive factors influencing type of acquired cold adaptation were discussed particularly focusing on the specific body temperature and thermoregulatory response experienced during the adaptation process. Finally, we presented practical implications based on this review. Despite recent advancements, further studies are needed to deepen our understanding of these complex adaptive processes and to identify the mechanisms that optimize cold adaptation across diverse environmental and physiological conditions.

## Abbreviations

ATP: Adenosine triphosphate
AVAs: Arteriovenous anastomoses
BMR: Basal metabolic rate
La: Blood lactate
BAT: Brown adipose tissue
CIT: Cold-induced thermogenesis
CIVD: Cold-induced vasodilation
CSR: Cold shock responses
CT: Computed tomography
*T*_co_: Core body temperature
*T*_cw_: Critical water temperature
cGMP: Cyclic guanosine monophosphate
Deoxy [Hb + Mb]: Deoxy hemoglobin and myoglobin
EMG: Electromyography
*T*_fing_: Finger skin temperature
FDG: Fluorodeoxy glucose
LT: Lactate threshold
VO_2max_: Maximal oxygen uptake
MVC: Maximal voluntary contraction
SUV_max_: Maximal standard uptake value
SUV_mean_: Mean standard uptake value
NPY: Neuropeptide Y
NO: Nitric oxide
NOS: Nitric oxide synthase
NFCI: Nonfreezing cold injury
NST: Non-shivering thermogenesis
NA: Noradrenaline
Oxy [Hb + Mb]: Oxy hemoglobin and myoglobin
PET: Positron emission tomography
PKG: Protein kinase G
ROS: Reactive oxygen species
RMR: Resting metabolic rate
ST: Shivering thermogenesis
SM: Skeletal muscle
SkBF: Skin blood flow
*I*_tissue_: Tissue insulation
StO_2_: Tissue oxygen saturation
Total [Hb + Mb]: Total hemoglobin and myoglobin
UCP1: Uncoupling protein 1
